# Health-related quality of life in adult patients with brain metastases after stereotactic radiosurgery: a systematic, narrative review

**DOI:** 10.1007/s00520-019-05136-x

**Published:** 2019-12-02

**Authors:** Eline Verhaak, Karin Gehring, Patrick E. J. Hanssens, Neil K. Aaronson, Margriet M. Sitskoorn

**Affiliations:** 1 Gamma Knife Center, Elisabeth-TweeSteden Hospital, Hilvarenbeekseweg 60, 5022 GC Tilburg, The Netherlands; 2 Department of Neurosurgery, Elisabeth-TweeSteden Hospital, Hilvarenbeekseweg 60, 5022 GC Tilburg, The Netherlands; 3 Department of Cognitive Neuropsychology, Tilburg University, Warandelaan 2, 5037 AB Tilburg, The Netherlands; 4 Division of Psychosocial Research and Epidemiology, Netherlands Cancer Institute, Plesmanlaan 121, 1066 CX Amsterdam, The Netherlands

**Keywords:** Brain metastases, Cancer, Health-related quality of life, Neoplasm metastasis, Patient-reported outcome measures, Radiosurgery

## Abstract

**Purpose:**

A growing number of patients with brain metastases (BM) are being treated with stereotactic radiosurgery (SRS), and the importance of evaluating the impact of SRS on the health-related quality of life (HRQoL) in these patients has been increasingly acknowledged. This systematic review summarizes the current knowledge about the HRQoL of patients with BM after SRS.

**Methods:**

We searched EMBASE, Medline Ovid, Web-of-Science, the Cochrane Database, PsycINFO Ovid, and Google Scholar up to November 15, 2018. Studies in patients with BM in which HRQoL was assessed before and after SRS and analyzed over time were included. Studies including populations of several types of brain cancer and/or several types of treatments were included if the results for patients with BM and treatment with SRS alone were described separately.

**Results:**

Out of 3638 published articles, 9 studies met the eligibility criteria and were included. In 4 out of 7 studies on group results, overall HRQoL of patients with BM remained stable after SRS. In small study samples of longer-term survivors, overall HRQoL remained stable up to 12 months post-SRS. Contradictory results were reported for physical and general/global HRQoL, which might be explained by the different questionnaires that were used.

**Conclusions:**

In general, SRS does not have significant negative effects on patients’ overall HRQoL over time. Future research is needed to analyze different aspects of HRQoL, differences in individual changes in HRQoL after SRS, and factors that influence these changes. These studies should take into account several methodological issues as discussed in this review.

**Electronic supplementary material:**

The online version of this article (10.1007/s00520-019-05136-x) contains supplementary material, which is available to authorized users.

## Background

Brain metastases (BM) originate from a malignancy outside the central nervous system. Most patients diagnosed with BM have primary lung cancer, breast cancer, or melanoma [1, 2]. Partly due to improved imaging such as MRI and improved systemic treatment of the primary cancer, the number of patients with BM is increasing [3–7].

Traditionally, most patients with BM have been treated with whole brain radiation therapy (WBRT) [3, 8, 9]. However, due to advances in the technology, and the increased availability, of stereotactic radiosurgery (SRS) and concerns about the long-term side effects of WBRT, radiation treatment is shifting toward SRS [3, 10–12]. The high precision of SRS spares healthy brain tissue, reducing the risks of long-term side effects [13, 14]. Although SRS is usually delivered in one fraction, it can be delivered in up to five fractions using a linear accelerator, particle beam accelerator or multisource Cobalt-60 unit [15].

Although the prognosis still remains poor [16–18], life expectancy in patients with BM is increasing due to improvements in systemic treatments of the primary tumor [6, 19]. Therefore, maintaining a good health-related quality of life (HRQoL) as long as possible is an important [20] primary objective in this patient group [21]. Consequently, management of BM is no longer focused solely on survival, but also on HRQoL and cognitive functioning of patients with BM after treatment [22–24].

Authors of previous clinical studies and reviews concluded that future trials that include patients with BM should assess HRQoL as outcome measure, to inform clinical practice (e.g., make informed treatment decisions, assess the efficacy of treatment, and inform patients about HRQoL over time) [21, 23–26]. In addition, HRQoL is important to evaluate as patients with BM rated HRQoL as the most important factor to be considered in choosing among available treatment options [27], as results from standard assessment of HRQoL in clinical practice may help communication between patients and clinicians [28], and as HRQoL appears to be an independent prognostic factor for survival [29–32].

To our knowledge, no systematic review has been conducted that focuses primarily on HRQoL outcomes after treatment with SRS alone in patients with BM. A synthesis of the available research findings can help to better understand patients’ HRQoL over time after SRS and can provide directions for future clinical trials. Ultimately, patients and physicians can be better informed on what to expect after SRS in terms of HRQoL. This systematic review summarizes the current knowledge on (changes in) the HRQoL of this patient group after SRS.

## Methods

### Literature search

A systematic literature search was conducted to identify studies in which adult patients with BM were treated with SRS, and HRQoL was assessed by means of a self-report questionnaire. EMBASE, Medline Ovid, Web-of-Science, the Cochrane Database, PsycINFO Ovid, and Google Scholar were searched up to November 15, 2018. Search terms were verified, and search strategies were built and performed by a biomedical information specialist of the library service of the Erasmus Medical Center, The Netherlands. Studies had to be published as empirical research articles in peer-reviewed journals and written in English, German, or Dutch. Case-report studies were excluded. Studies with an HRQoL assessment before and at least one HRQoL assessment after SRS alone were included. Within-group analyses had to be performed on HRQoL data. Studies that included a heterogeneous sample of patients in terms of type of brain-involved malignancies and/or studies in which different types of treatment were evaluated, were included only if the results for patients with BM treated with SRS alone were reported separately. Inclusion and exclusion criteria in terms of PICOs (patient, intervention, comparison, outcome) and search strategies are presented in Online Resource 1.

### Study selection

All studies were screened by the first author (E.V.) based on title and abstract. If eligibility was not clear from the title and abstract, the full text was screened. Papers that potentially met the eligibility criteria after full text screening were also reviewed by the second author (K.G.). Consensus was reached in all cases. This review is a qualitative synthesis of empirical studies. The same two authors extracted data from the included studies and results were compared; there were no disagreements. Reference lists of eligible articles were screened for additional articles.

### Assessment of included studies

Factors that were cross-checked and critically evaluated among the studies included the following: type of cohorts/study samples included (e.g., different histologies of primary cancers), prior BM treatment, compliance or reasons for dropout reported, primary endpoints, HRQoL questionnaire used, timing of baseline HRQoL assessment, and timing and place of post-measurements.

## Results

### Selected studies

**Fig. 1:**
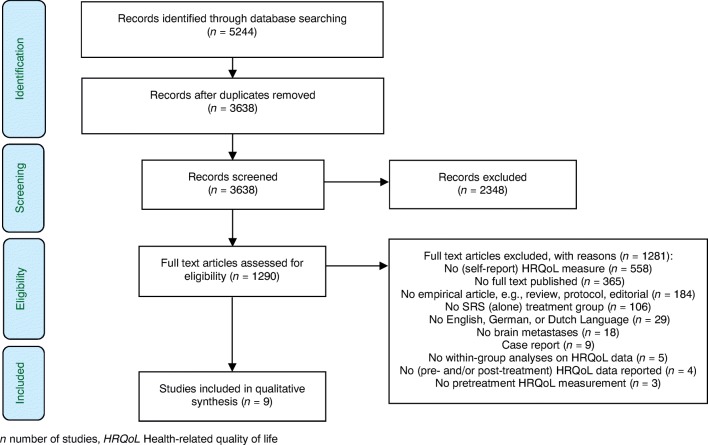
PRISMA flowchart of the study selection

The systematic literature search identified 3638 unique records (Fig. 1). After screening title and abstract, 1290 full texts were considered, and ultimately 9 studies were included in the review (Table 1).

**Table 1:** Prospective studies of the HRQoL of patients with BM undergoing SRS

First author, year Study design Modality (dose)	Participants (*n*), median (range)	Histology (*n*)	Primary endpoint	HRQoL questionnaire Scales	HRQoL after SRS^b^	Comments
Studies using brain cancer–specific HRQoL questionnaires						
Skeie, 2017 [33] Single-arm study Gamma knife (mean 22.7; 15–25 Gy)	97 patients with 1–6 BM Age 64 (32–86) Female (51) Mean BM volume 6.3 cm^3^ (0.072–25.2) KPS 80 (60–100)^a^	Lung (45) Colorectal (17) Skin (12) Other (23)	Health-related quality of life	FACT-Br All 8 scales	Group level: stable HRQoL after SRS up to 12 months, except for general HRQoL. Individual level: less or stable additional concerns (brain cancer subscale) for 64%, 60%, 66%, 72%, and 60% of the patients at respectively 1, 3, 6, 9, and 12 months (based on MCIDs; cutoff point unclear).	Prior treatment with the following: WBRT (*n* = 9), surgery (*n* = 6), surgery and WBRT (*n* = 5). Follow-up questionnaires sent by mail.
Bragstad, 2017 [32] Single-arm study Gamma knife (mean 40.4; 16–25 Gy)	44 patients with 1–5 BM Age 62.8 (42–82) Female (19) KPS 100 (5); 90 (14); 80 (9); 70 (10); < 70 (6) Steroid use at GKRS (34)	NSCLC (39) SCLC (5)	Health-related quality of life	FACT-Br All 8 scales	Group level: stable HRQoL up to 12 months after SRS Individual level: at 1 month (*n* = 39), 23 patients had less, 6 had stable, and 9 had more additional concerns (brain cancer subscale).	Only patients with primary lung cancer included. Prior treatment with the following: surgery, WBRT, or surgery and WBRT (number of patients not reported). Reasons for dropout not reported. Follow-up questionnaires sent by mail.
Chang, 2007 [34] Single-arm study (pilot) Linear accelerator (median 20; 15–24 Gy)	15 patients with 1–3 BM Age 64.9 (31.5–77) Female (10) Total BM volume 1.76 cm^3^ (0.16–19.98) KPS 90 (70–100)^a^	NSCLC (8) Melanoma (4) Renal cell (3)	Cognitive functioning	FACT-Br One preselected total score; FACT-Brain score	Individual level: 11 patients with stable and 2 patients with declined HRQoL scores 1 month after SRS (based on the RCI). For 4 out of 5 long-term survivors (patients with an evaluation ≥ 200 days after SRS) scores remained relatively stable up to their last evaluation after SRS (± 8.5 months); a decline was observed in a patient who was hospitalized because of edema.	Measurement of pretreatment HRQoL after SRS in an unknown number of patients. Reasons for dropout not reported. No report of mean HRQoL scores at all time points.
Kirkpatrick, 2015 [35] Single-arm study (randomized per lesion) Linear accelerator (15–24 Gy)	49 patients with 1–3 BM Age 61 (26–87) Female (33) KPS 90 (70–100)^a^ Chemotherapy before SRS (28) Chemotherapy after SRS (43)	NSCLC (25) Melanoma (8) Breast (6) Other (10)	Local recurrence	FACT-Br All 8 scales	Group level: stable HRQoL 3 months after SRS (*n* = 24).	Reasons for dropout not reported. Poor compliance with HRQoL assessment. Randomized per lesion to a 1- or 3-mm margin expansion group.
Habets, 2016 [36] Single-arm study Linear accelerator (18–24 Gy)	97 patients with 1–4 BM Mean age 63 (33–82) Female (51) Total BM volume 7.8 cm^3^ (0.12–63.9) KPS 80 (60–100) Chemotherapy < 3 months of SRS (13) Use of corticosteroids (85) Use of anti-epileptic drugs (21)	NSCLC (48) Renal (12) Melanoma (9) Other (28)	Cognitive functioning	EORTC-QLQ-C30 All 9 scales and 6 single items EORTC-QLQ-BN20 All 4 scales and 7 single items	Group level: worse scores on physical functioning, nausea, appetite loss, and (more bothered by) hair loss over 6 months’ time after SRS. Other aspects of HRQoL were stable over time.	No report of mean HRQoL scores at follow-up. Patients treated with WBRT during the study were no longer followed (*n* = 13). BM with volumes > 13 cm^3^ or near the brainstem were treated in 3 fractions of 8 Gy; other BM were treated in 1 fraction of 18–21 Gy.
van der Meer, 2018 [37] Single-arm study Linear accelerator (18–24 Gy)	55 patients with 1–4 BM Mean age 63, SD 9 Female (30) Total BM volume 7.3 cm^3^ (0.12–63.9) KPS 80 (interquartile range 80–90) Chemotherapy (6) Use of corticosteroids (48) Use of anti-epileptic drugs (12)	NSCLC (27) Renal (11) Melanoma (4) Other (13)	Cognitive functioning	EORTC-QLQ-C30 6 preselected scales; global health status, physical, emotional, role, cognitive functioning, and fatigue EORTC-QLQ-BN20 2 preselected scales; motor dysfunction and communication deficits	Individual level, scale: at 3 months, on 4 out of 8 HRQoL scales, most patients had stable scores; on 3 scales, most patients had a decline in scores; and on 1 scale, most patients had improved scores. At 6 months, on 7 HRQoL scales, most patients had stable scores; and on 1 scale, most patients had worse scores (based on an increase or decrease of ≥ 10 points). Individual level, patient: after 3 and 6 months, 22% and 21% of patients reported a decline on at least one HRQoL scale (other scales declined as well or remained stable), 12% and 18% an improvement on at least one HRQoL scale, 64% and 58% both a decline as an improvement, and 2% and 3% had stable scores on all HRQoL scales, respectively (based on an increase or decrease of ≥ 10 points).	No report of mean HRQoL scores at all time points. Patients treated with WBRT during the study were no longer followed (number of patients not reported). BM with volumes > 13 cm^3^ or near the brainstem were treated in 3 fractions of 8 Gy; other BM were treated in 1 fraction of 18–21 Gy.
Studies using generic HRQoL questionnaires						
Miller, 2017 [38] Single-arm Gamma knife (median 22; 20–24 Gy)	67 patients Mean age 59, SD 11 Female (37) KPS 80 (70–90) Chemotherapy in past month (67)	NSCLC (30) Breast (14) Melanoma (9) Other (14)	Time to health state (EQ-5D index) failure	EQ-5D All 5 dimensions	Group level: worse scores on all dimensions at patients’ last follow-up (analyzed on the basis of 122 treatments) Individual level: overall health state failure in 28% and improvement in 24% of treatments (MCID of 0.1) and self-perceived health state failure in 50% and improvement in 41% of treatments (MCID of 10).	Limited patient characteristics (mostly per treatment). Prior treatment with the following: WBRT (*n* = 41) and resection (*n* = 21). Reasons for dropout not reported. Measurement of pretreatment HRQoL after SRS in an unknown number of patients. Mean HRQoL scores only reported at last follow-up. Only HRQoL data at a patients’ last follow-up was analyzed. HRQoL data analyzed per treatment.
Kotecha, 2017 [39] Single-arm study Gamma knife (15–24 Gy)	59 patients with 1–11 BM Baseline characteristics of the 27 patients with HRQoL assessment not reported	NA	Intracranial recurrence patterns after multiple SRS courses	EQ-5D All 5 dimensions	Group level: longitudinal overall health state remained relatively stable over time. Worse scores on mobility, self-care, usual activities, overall health state, and self-perceived health state at patients’ last follow-up (data abstracted from a table). Individual level: at patients’ last follow-up, most patients declined (48%, 54%) or improved (45%, 45%) on overall health state and self-perceived health state, respectively, based on the MCIDs (data abstracted from a table). At 1 year, most patients were free from HRQoL failure (overall health state 77%; self-perceived health state 69%).	patients who underwent a minimum of 3 SRS courses for BM were included. Prior treatment with the following: WBRT (*n* = 19). Reasons for dropout not reported. Mean HRQoL scores only reported at last follow-up. Only HRQoL data at a patients’ last follow-up was analyzed.
Randolph, 2017 [40] Single-arm study Gamma knife (median 20; 10–24 Gy)	114 patients with 1–4 BM Baseline characteristics of the 39 patients with HRQoL assessment not reported	NA	Local control Distant control Overall survival	SQLI Total score	Group level: significant decline in HRQoL scores 6 and 12 months after SRS compared with baseline No significant difference between 6 and 12 months after SRS.	Subgroup of geriatric patients (age ≥ 70). Previous treatments not reported. Reasons for dropout not reported.

*BM* brain metastases, *EORTC-QLQ-BN20* European Organization for Research and Treatment of Cancer Quality of Life Questionnaire Brain Cancer Module, *EORTC-QLQ-C15-PAL* European Organization for Research and Treatment of Cancer Quality of Life Questionnaire Core 15 Palliative care, *EORTC-QLQ-C30* European Organization for Research and Treatment of Cancer Quality of Life Questionnaire Core 30, *EQ-5D* EuroQol 5 Dimensions, *FACT-Br* Functional Assessment of Cancer Therapy-Brain, *HRQoL* health-related quality of life, *KPS* Karnofsky Performance Status, *MCID* minimum clinically importance difference, *n* number of patients, *NA* not applicable, *NSCLC* non–small cell lung cancer, *RCI* reliable change index, *SCLC* small cell lung cancer, *SD* standard deviation, *SQLI* Spitzer Quality of Life Index, *SRS* stereotactic radiosurgery, *WBRT* whole brain radiation therapy

^a^Calculated from data presented in table with patient characteristics, ^b^Group level: analyses of mean scores of the total group; individual level: number/percentage of patients with deviant scores according to a normative threshold/cutoff

### Characteristics of studies

All studies included a heterogeneous group of patients with different primary histologies, except for one study [32], in which only patients with primary lung cancer were included. In one study [40], only geriatric patients (age ≥ 70) were included. In one other study [39], patients were included who already received 3 courses of SRS, whereas in most studies, patients were included before their first course of SRS. Baseline characteristics of patients with baseline HRQoL scores were not reported in two studies [39, 40]. For two studies [33, 36], a proportion of patients was also included in a subsequent study (respectively [32, 37]). Sample sizes in the 9 selected studies ranged from 15 to 97 patients. In most studies, patients were female (range 43.2 to 67.3%), had primary lung cancer (range 37.3 to 100%), and had a median Karnofsky Performance Status (KPS) score of 80 (range < 70 to 100) (Table 1). In four studies [34–37], patients with newly diagnosed BM were included; in four other studies [32, 33, 38, 39], patients received prior BM treatment; and in one study [40], it was not reported if patients received prior BM treatment. Reasons for dropout were not reported in 6 studies [32, 34, 35, 38–40], and in two studies [36, 37], reasons of dropout were reported, but without the numbers of patients (Table 1).

### HRQoL assessments

Results on HRQoL over time of all reviewed studies are presented in Table 1. In three [32, 33, 38] out of nine studies, HRQoL was the primary outcome measure. Four studies [32, 33, 38, 39] evaluated HRQoL both at the group level and at the individual level, two studies [35, 36] evaluated HRQoL at the group level only, and two studies [34, 37] evaluated HRQoL at the individual level only. In the studies reviewed, HRQoL was measured with 5 different self-report questionnaires. The most frequently used questionnaire was the brain cancer–specific Functional Assessment of Cancer Therapy Brain (FACT-Br), used in 4 studies (Table 2). The most commonly investigated aspects of HRQoL at the group level were physical, general/global, social, and emotional aspects. In six studies [32–37], cancer-specific HRQoL self-report questionnaires were used to measure HRQoL, and in three studies [38–40], generic HRQoL self-report questionnaires were used to measure HRQoL. In two studies [34, 38], an unknown number of patients completed the “pretreatment”/baseline HRQoL measurement after SRS. Follow-up questionnaires were sent by mail in two studies [32, 33], and in the other studies, administration was scheduled to coincide with hospital visits after SRS. In five studies [34, 36–39], mean HRQoL scores during follow-up were not reported, although in two of them [38, 39], mean HRQoL at patients’ last follow-up were reported (this point is not the same for each patient).

**Table 2:** HRQoL questionnaires

HRQoL instrument	Description	Scales/items	Used by
Functional Assessment of Cancer Therapy-Brain (FACT-Br)	The FACT-Br was developed for patients with primary brain tumors. Questions are answered on a 5-point Likert scale ranging from 0 (not at all) to 4 (very much). The FACT-Br consists of 5 subscales, 2 total scales, and 1 index. The FACT-General is a summary of general HRQoL and can be used in diverse patient groups. The FACT-Br combines the FACT-G with a disease-specific subscale score for patients with a brain tumor. The TOI is assumed to be more responsive to change after treatment than a total HRQoL score. Higher scores on each (sub)scale indicate better health-related quality of life [41–43]. The FACT-Br has high validity and reliability coefficients [42] and is a valid measure for use in patients with brain metastases [43].	• Five subscales o Physical well-being o Social/family well-being o Emotional well-being o Functional well-being o Brain cancer subscale (additional concerns specific for patients with brain tumors) • Two total scales o FACT-General (FACT-G; physical + social + emotional + functional well-being) o FACT-Brain (FACT-BR; FACT-G + brain cancer subscale) • One index o Trial Outcome Index (TOI; physical + functional well-being + brain cancer subscale)	Chang, 2007 [34] Kirkpatrick, 2015 [35] Skeie, 2017 [33] Bragstad, 2017 [32]
European Organization for Research and Treatment of Cancer Quality of Life Questionnaire Core 30 (EORTC-QLQ-C30)	The EORTC QLQ-C30 consists of 5 functional scales, 3 symptom scales, 1 global health/quality of life scale and 6 single items. All of the scales and single-item measures range in scores from 0 to 100, with higher scores reflecting more severe symptoms. In case of functional scales, higher scores reflect better functioning. The EORTC QLQ-C30 is a reliable and valid measure of the quality of life of patients with cancer [44].	• Five functional scales o Physical functioning o Role functioning o Cognitive functioning o Emotional functioning o Social functioning • Three symptom scales o Fatigue o Pain o Nausea and vomiting • One global quality of life scale • Six single items (dyspnea, insomnia, appetite loss, constipation, diarrhea, financial difficulties)	Habets, 2016 [36] van der Meer, 2018 [37]
EORTC-QLQ Brain Cancer Module (EORTC-QLQ-BN20)	The EORTC-QLQ-BN20 was developed for brain cancer patients and is designed to complement the QLQ-C30. It consists of 20 items and four subscales. All items and scale scores are linearly transformed to a 0–100 scale, with higher scores reflecting more severe symptoms. The BN20 has adequate psychometric properties for use in assessing the HRQoL of brain cancer patients in international studies [45, 46].	• Four subscales o Future uncertainty o Motor dysfunction o Visual disorder o Communication deficit • Seven single items (headaches, hair loss, weakness of legs, seizures, itchy skin, bladder control, drowsiness)	Habets, 2016 [36] van der Meer, 2018 [37]
Spitzer Quality of Life Index (SQLI)	The Spitzer Quality of Life Index was developed for use by physicians to assess the relative benefits and risks of various treatments for serious illness. It consists of 5 questions concerning HRQoL according to five factors. For each item, a score of 0, 1, or 2 is obtained; the maximum score is 10. Lower scores reflect better performance. The SQLI has convergent discriminant and content validity among cancer patients [47].	• One index consisting of 5 factors o Activity o Support o Daily living o Outlook o Health	Randolph, 2017 [40]
EuroQol 5 Dimensions questionnaire (EQ-5D)	The EQ-5D was developed as standardized measure of health state, applicable to a wide range of patient populations. It consists of 5 items representing 5 dimensions. Each item is answered on a 3-point scale; 1 no problems, 2 some problems, and 3 extreme problems. The index score overall health state consists of all 5 items and ranges between 0 (dead) and 1 (best possible health). Self-perceived health state is measured on a 20-cm vertical scale with endpoints 0 (worst imaginable health) and 100 (best imaginable health) [48–50].	• Five subscales/dimensions o Mobility o Self-care o Usual activities o Pain/discomfort o Anxiety/depression • One index o Overall health state (all 5 subscales) • One vertical visual analogue scale o Self-perceived health state	Kotecha, 2017 [39] Miller, 2017 [38]

*FACT-Br* Functional Assessment of Cancer Therapy-Brain, *FACT-G* FACT-General, *TOI* Trial Outcome Index, *EORTC-QLQ-C30* European Organization for Research and Treatment of Cancer Quality of Life Questionnaire Core 30, *EORTC-QLQ-BN20* European Organization for Research and Treatment of Cancer Quality of Life Questionnaire Brain Cancer Module, *SQLI* Spitzer Quality of Life Index, *EQ-5D* EuroQol 5 Dimensions

## Discussion

The aim of this review was to summarize findings of studies on (changes in) the HRQoL of patients with BM after SRS. Nine studies were included. Conclusions on HRQoL after SRS however should be drawn with caution, as several (methodological) limitations (discussed below) complicate the interpretation of findings. In two studies on individual scores only, stable overall HRQoL was demonstrated in most patients [34, 37]. In four out of seven studies evaluating group scores, overall HRQoL remained stable in patients with BM after SRS [32, 33, 35, 36], even up to 12 months after SRS in small groups of long-term survivors [32, 33]. However, the three other studies found a decline in overall HRQoL after SRS. One of these studies reported a decline in overall HRQoL 6 and 12 months after treatment in an otherwise undefined small subgroup of geriatric patients (age ≥ 70) [40], and two other studies reported a statistically significant decline in overall HRQoL at patients’ last follow-up [38, 39].

These last two studies [38, 39] most likely assessed HRQoL at the point of progressive disease for many patients, as no further follow-up assessments could be completed. As several studies report a decline in HRQoL after progressive disease [31, 33, 36, 41, 42], the occurrence of progressive disease might explain why these two studies found a decline in HRQoL while other studies reported stable HRQoL during multiple follow-up assessments. Differences in negative and stable outcomes might also be due to different patient or treatment characteristics. In one of these studies [39], patients underwent a minimum of three SRS courses before inclusion and patient characteristics were not reported. However, baseline patient and treatment characteristics in the other study [38] were comparable with the baseline characteristics in the studies reporting stable HRQoL after SRS [32, 33, 35, 36].

Although HRQoL scores on the group level appear to remain stable over time, they may mask individual changes in HRQoL. Habets et al. [36] reported stable HRQoL over time on the group level, while analysis of individual results from a portion of the same study sample on a selection of the scales by van der Meer et al. [37] showed that most patients demonstrated both improvements as well as deterioration in different aspects of HRQoL over time. Four other studies [32, 33, 38, 39] evaluated both group and individual changes in HRQoL after SRS. Two studies found stable mean group scores on *additional concerns* over time, while on the individual level, the majority of patients (60%) reported less *additional concerns* [32] and small groups of patients (23 to 36%) reported more *additional concerns* 1 month after SRS [32, 33]. Two other studies that investigated HRQoL at patients’ last follow-up (median HRQoL follow-up 12 and 19 months) found a decline in group scores on *overall health state* and *self-perceived health state*, whereas on the individual level, similar and substantial percentages of patients improved (*overall health state*, 24% versus 45%; and *self-perceived health state*, 41% versus 45%) and declined (*overall health state*, 28% versus 48%; and *self-perceived health state*, 50% versus 54%) [38, 39]. Differences in the percentages of improved and declined *overall health state*s between both studies may be explained by chance due to the small sample size (*n* = 27) in one of these studies [39]; in addition, patients in this study had already undergone a minimum of three SRS courses before inclusion in the study.

Similarly, combining the multidimensional aspects of HRQoL, including physical, social, and emotional functioning [43], into a single overall HRQoL score may also lead to a loss of information or mask potential improvements and declines in more specific aspects of HRQoL. One study [34] evaluated an overall HRQoL score only, limiting conclusions about the full range of potentially different HRQoL effects. However, preselecting certain HRQoL subscales based on existing literature and/or clinical insights is a more conservative approach than assessing a wide range of HRQoL outcomes which might lead to potential problems with type I errors in statistical testing due to multiple comparisons.

At the group level, the most frequently evaluated aspects of HRQoL were physical, general/global, social, and emotional aspects. Mean scores of these aspects remained stable over time [32, 33, 35, 36], except for physical well-being/functioning and general/global HRQoL. On these aspects, contradictory results were reported. Three studies using the EORTC-QLQ-C30 or EQ-5D reported a decline in the physical aspect of HRQoL [36, 38, 39], while 3 other studies using the FACT-Br reported stable scores over time [32, 33, 35]. This can be explained by the different questionnaires that were used. For example, the subscale *physical functioning* of the EORTC-QLQ-C30 and the subscales *mobility*, *self-care*, and *usual-activities* of the EQ-5D are more focused on physical *activities*, while the subscale *physical well-being* of the FACT-Br is more focused on physical *symptoms*. It should be noted in addition that declines were reported by the two studies in which HRQoL was assessed at a patients’ last follow-up. This might also explain the difference in findings among studies on general/global HRQoL; the two studies measuring HRQoL at patients’ last follow-up reported a decline [38, 39], while four other studies reported stable scores [32, 33, 35, 36]. However, the different setup in questionnaires might also play a role. Since there is no standard assessment tool for HRQoL in patients with BM, comparing results from studies using different HRQoL measurements remains a challenge [23, 24].

It should be noted that in two studies [34, 38] an unknown number of patients completed the pretreatment HRQoL measurement after SRS, which may have affected conclusions on HRQoL over time. In addition, in five studies [34–37, 40], previous treatments directed at the BM could have negatively affected the HRQoL of the patients. In two studies [32, 33], follow-up questionnaires were sent by mail; consequently, it was not known whether patients completed the self-report questionnaire themselves without the influence of significant others. On the other hand, patients could fill out these questionnaires at home, which may cause less stress or anxiety compared with the other studies, in which questionnaires were administered in the hospital at control visits, and thus provide a more realistic representation of HRQoL in daily life.

Among the other methodological limitations of studies on HRQoL after SRS was the lack of (reporting of) within-group analyses. To investigate changes in HRQoL after SRS, within-group analyses are needed to be able to draw conclusions on the effect of SRS on HRQoL over time. Unfortunately, several studies did not perform such analyses or did not report the results [31, 42, 44–46] and were therefore not included in this review.

When interpreting results from longitudinal studies on HRQoL after SRS, it is important to be aware that a range of other factors, besides the treatment of interest, may influence HRQoL over time, including medication use (e.g., steroids), effects of treatment for the primary tumor (including chemotherapy, immunotherapy, radiation, surgery), pseudo-progression or progression of disease, HRQoL before treatment, cognitive symptoms, and the mere passage of time. For example, low mood after the diagnosis of BM may be alleviated by the use of an antidepressant or just passage of time. In four of the included studies, factors that affected (changes in) HRQoL after SRS were evaluated. These studies suggested that HRQoL after SRS was associated with KPS, total tumor volume in the brain, symptomatic BM, time since SRS, and disease progression (e.g., intra- and extracranial tumor activity) [32, 33, 36, 38], while the number of BM, sex, and age did not appear to influence HRQoL [32, 33, 36]. However, due to differences between these studies in statistical techniques employed (univariate and multivariate), differences in the choice as to which predictors were investigated and at which time points, it was not possible to draw reliable conclusions.

In addition, a potential effect of “response shift” should be considered. A response shift refers to changes in patients’ internal standards, values, and conceptualization of HRQoL that may occur during the course of their disease [47–50]. Studies have shown that although the clinical health status of patients with cancer might deteriorate considerably over time, HRQoL scores often remain stable [47]. Most of the studies reviewed did not find considerable deterioration of HRQoL, which may be (partly) explained by the response shift phenomenon. However, although patients might have shifted their response pattern over time, their self-reported HRQoL may still reflect their actual *personal interpretation* of their HRQoL at a given point in time [51].

High attrition and low response rates are very common in studies that include patients whose life expectancy is short [24, 48, 52]. In many of the studies reviewed, the number of patients completing (long-term) follow-up assessments dropped substantially. In most studies, reasons for dropout (e.g., decease, disease progression, personal motivation) were not or only partly described [32, 34–40]. As a result, interpretation of results is complicated [48], and results might not be generalizable to the whole population of patients [53]. However, if the reasons for dropout are related to the disease (progression or death), rather than personal motivation, the results still are very informative with respect to the subgroup of patients who survive on the longer term. Reporting the reasons for dropout is therefore very important for proper interpretation of study results.

The timing of follow-up measurements varied across the studies reviewed and only 3 studies [32, 33, 40] had follow-up periods longer than 6 months; in two other studies [38, 39], HRQoL was assessed at last follow-up, which differed for each patient.

Several limitations of the review process should be noted as well. Abstract screening was carried out by only one author, and thus, we cannot rule out the possibility that one or more additional relevant articles might have been identified if another author had been involved in this screening process. However, we believe that the screening process as carried out was very thorough. It is also possible that relevant studies were excluded due to language constraints. A risk of publication bias cannot be ruled out, since, for example, gray literature was not included in this review.

### Future research

The synthesis of the findings of the nine relevant studies revealed that future clinical trials on the effects of SRS on HRQoL of patients with BM are needed to further investigate the multiple aspects of HRQoL over time, individual changes in HRQoL after treatment, and factors that influence HRQoL. Studies should report within-group changes and clearly describe statistical analyses and reasons for dropout. For the assessment of HRQoL in this patient population, brain cancer–specific self-report HRQoL questionnaires, evaluating the different aspects of HRQoL, should be used. To minimize patient burden and therefore prevent high dropout rates, dedicated personnel should be available to administer HRQoL questionnaires, and follow-up HRQoL assessments should be scheduled to coincide with and take place before, instead of after, standard hospital visits after SRS [48, 54]. In addition, more studies with adequate sample sizes at long-term follow-ups (e.g., > 6 months) are needed to analyze different aspects of HRQoL at these time points, especially because irreversible and progressive radiation-induced brain injury, including cognitive impairment, usually emerges > 6 months after treatment [55, 56]. There are many methodological and logistical challenges in performing serial HRQoL assessments in these patients, but the payoff in terms of increased understanding of the effect of both the disease and its treatment on the functional health, symptom burden, and well-being of our patients justifies the additional investment required.

### Relevance for clinical practice

HRQoL appears to be an independent prognostic factor for survival in cancer patients with and without BM [29–32], and in a recent study [27], HRQoL was rated by patients with BM as the most important factor to be considered in choosing among available treatment options. Since more patients with multiple BM are treated with SRS, it is important to know how this treatment may affect the HRQoL of patients over time. In general, results of the studies reviewed here suggest that SRS does not have a significant negative effect on patients’ overall HRQoL over time (even up to 12 months after SRS). This indicates that, in terms of HRQoL, SRS can be safely used in the management of patients with BM. Although more research is needed on factors influencing HRQoL of patients with BM, the current evidence suggests that clinicians should pay additional attention to patients with low KPS, large tumor volumes, symptomatic BM, and disease progression. In addition, assessment of HRQoL in clinical practice may improve communication between patients and clinicians, is helpful to identify patients’ concerns [28], and helps clinicians to provide patients with personalized information. This emphasizes the importance of incorporating HRQoL measures as a standard part of clinical care in patients with BM.

## Electronic supplementary material

ESM 1 (DOCX 21 kb)
