# Supplementary material for: Health-related quality of life in adult patients with brain metastases after stereotactic radiosurgery: a systematic, narrative review
Source: Support Care Cancer. 2019 Dec 2;28(2):473–84. doi: 10.1007/s00520-019-05136-x (PMC6954134; doi:10.1007/s00520-019-05136-x)
Supplement: Supplementary file 1 — (DOCX 21 kb) [file 520_2019_5136_MOESM1_ESM.docx]

# Health-related quality of life in adult patients with brain metastases after stereotactic radiosurgery: a systematic, narrative review

Journal: Supportive Care in Cancer

**Eline Verhaak, MSc^1,2,3^, Karin Gehring, PhD^2,3^, Patrick E. J. Hanssens, MD^1,2^, Neil K. Aaronson, PhD^4^, AND Margriet M. Sitskoorn, PhD^2,3^**

^1^ Elisabeth-TweeSteden Hospital, Gamma Knife Center, Hilvarenbeekseweg 60, 5022 GC, Tilburg, The Netherlands

^2^ Elisabeth-TweeSteden Hospital, Department of Neurosurgery, Hilvarenbeekseweg 60, 5022 GC, Tilburg, The Netherlands

^3^ Tilburg University, Department of Cognitive Neuropsychology, Warandelaan 2, 5037 AB, Tilburg, The Netherlands

^4^ Netherlands Cancer Institute, Division of Psychosocial Research and Epidemiology, Plesmanlaan 121, 1066 CX, Amsterdam, The Netherlands

**Corresponding author:** Karin Gehring (E-mail: k.gehring@tilburguniversity.edu, Telephone number: +31 13 466 4233)

# Online Resource 1

**Table 1**. Inclusion and exclusion criteria in terms of PICOS

| Patients | Adult patients with at least 1 BM  Any primary cancer  No restriction for prognosis, country or clinical setting |
| --- | --- |
| Intervention | Stereotactic radiosurgery (only) |
| Comparison | Change in HRQoL after stereotactic radiosurgery (within-group analyses) |
| Outcomes | Self-report HRQoL questionnaire |

Search strategies

**Embase.com**

('brain tumor'/exp OR (brain/exp AND metastasis/exp) OR (((brain OR intracereb* OR cerebr* OR intracrani* OR Infratentor* OR skull) NEAR/3 (tumor* OR tumour* OR neoplas* OR metasta* OR malign*))):ab,ti) AND (Radiosurgery/exp OR 'stereotactic procedure'/de OR 'stereotactic radiosurgery'/de OR 'gamma knife'/exp OR (Radiosurg* OR (Radio NEXT/1 surg*) OR 'gamma knife' OR gammaknife OR CyberKnife OR (Fractionat* NEAR/3 radiotherap*) OR GKSR OR stereotactic* OR stereotaxic*):ab,ti) AND ('quality of life'/exp OR 'mood disorder'/exp OR 'emotion'/exp OR 'fatigue'/exp OR 'mental health'/exp OR 'psychological aspect'/de OR 'functional assessment'/exp OR 'functional status assessment'/exp OR 'functional status'/exp OR 'stress'/exp OR ((qualit* NEAR/3 life) OR hrql OR hrqol OR qol OR depressi* OR (mood NEAR/3 disorder*) OR affect* OR emotion* OR fatigue* OR (mental* NEAR/3 health*) OR well-being* OR wellbeing* OR Anxi* OR psycholog* OR ((functional* OR mental*) NEAR/3 (assess* OR status OR state)) OR EORTC OR 'Rotterdam Symptom* Checklist*' OR rscl OR stress OR distress OR (('Short Form' OR sf) NEXT/1 (36 OR 20 OR 12 OR 8)) OR sf36 OR sf20 OR sf12 OR sf8 OR 'Anderson Symptom* Inventor*' OR MDASI OR hads OR BCM20 OR 'Brain Cancer Module'):ab,ti)

**Medline Ovid**

(exp "Brain Neoplasms"/ OR (brain/ AND "Neoplasm Metastasis"/) OR (((brain OR intracereb* OR cerebr* OR intracrani* OR Infratentor* OR skull) ADJ3 (tumor* OR tumour* OR neoplas* OR metasta* OR malign*))).ab,ti.) AND (exp "Stereotaxic Techniques"/ OR (Radiosurg* OR (Radio ADJ surg*) OR "gamma knife" OR gammaknife OR CyberKnife OR (Fractionat* ADJ3 radiotherap*) OR GKSR OR stereotactic* OR stereotaxic*).ab,ti.) AND ("quality of life"/ OR exp "Mood Disorders"/ OR exp "emotions"/ OR exp "fatigue"/ OR "mental health"/ OR psychology.xs. OR "Stress, Psychological"/ OR ((qualit* ADJ3 life) OR hrql OR hrqol OR qol OR depressi* OR (mood ADJ3 disorder*) OR affect* OR emotion* OR fatigue* OR (mental* ADJ3 health*) OR well-being* OR wellbeing* OR Anxi* OR psycholog* OR ((functional* OR mental*) ADJ3 (assess* OR status OR state)) OR EORTC OR "Rotterdam Symptom* Checklist*" OR rscl OR stress OR distress OR (("Short Form" OR sf) ADJ (36 OR 20 OR 12 OR 8)) OR sf36 OR sf20 OR sf12 OR sf8 OR "Anderson Symptom* Inventor*" OR MDASI OR hads OR BCM20 OR "Brain Cancer Module").ab,ti.)

**psycINFO Ovid**

(exp "Brain Neoplasms"/ OR (((brain OR intracereb* OR cerebr* OR intracrani* OR Infratentor* OR skull) ADJ3 (tumor* OR tumour* OR neoplas* OR metasta* OR malign*))).ab,ti.) AND ("Stereotaxic Techniques"/ OR (Radiosurg* OR (Radio ADJ surg*) OR "gamma knife" OR gammaknife OR CyberKnife OR (Fractionat* ADJ3 radiotherap*) OR GKSR OR stereotactic* OR stereotaxic*).ab,ti.) AND ("quality of life"/ OR exp "Affective Disorders"/ OR exp "emotions"/ OR exp "fatigue"/ OR "mental health"/ OR "Stress"/ OR ((qualit* ADJ3 life) OR hrql OR hrqol OR qol OR depressi* OR (mood ADJ3 disorder*) OR affect* OR emotion* OR fatigue* OR (mental* ADJ3 health*) OR well-being* OR wellbeing* OR Anxi* OR psycholog* OR ((functional* OR mental*) ADJ3 (assess* OR status OR state)) OR EORTC OR "Rotterdam Symptom* Checklist*" OR rscl OR stress OR distress OR (("Short Form" OR sf) ADJ (36 OR 20 OR 12 OR 8)) OR sf36 OR sf20 OR sf12 OR sf8 OR "Anderson Symptom* Inventor*" OR MDASI OR hads OR BCM20 OR "Brain Cancer Module").ab,ti.)

**Cochrane**

((((brain OR intracereb* OR cerebr* OR intracrani* OR Infratentor* OR skull) NEAR/3 (tumor* OR tumour* OR neoplas* OR metasta* OR malign*))):ab,ti) AND ((Radiosurg* OR (Radio NEXT/1 surg*) OR 'gamma knife' OR gammaknife OR CyberKnife OR (Fractionat* NEAR/3 radiotherap*) OR GKSR OR stereotactic* OR stereotaxic*):ab,ti) AND (((qualit* NEAR/3 life) OR hrql OR hrqol OR qol OR depressi* OR (mood NEAR/3 disorder*) OR affect* OR emotion* OR fatigue* OR (mental* NEAR/3 health*) OR well-being* OR wellbeing* OR Anxi* OR psycholog* OR ((functional* OR mental*) NEAR/3 (assess* OR status OR state)) OR EORTC OR 'Rotterdam Symptom* Checklist*' OR rscl OR stress OR distress OR (('Short Form' OR sf) NEXT/1 (36 OR 20 OR 12 OR 8)) OR sf36 OR sf20 OR sf12 OR sf8 OR 'Anderson Symptom* Inventor*' OR MDASI OR hads OR BCM20 OR 'Brain Cancer Module'):ab,ti)

**Web-of-Science**

TS=(((((brain OR intracereb* OR cerebr* OR intracrani* OR Infratentor* OR skull) NEAR/2 (tumor* OR tumour* OR neoplas* OR metasta* OR malign*)))) AND ((Radiosurg* OR (Radio NEAR/1 surg*) OR "gamma knife" OR gammaknife OR CyberKnife OR (Fractionat* NEAR/2 radiotherap*) OR GKSR OR stereotactic* OR stereotaxic*)) AND (((qualit* NEAR/2 life) OR hrql OR hrqol OR qol OR depressi* OR (mood NEAR/2 disorder*) OR affect* OR emotion* OR fatigue* OR (mental* NEAR/2 health*) OR well-being* OR wellbeing* OR Anxi* OR psycholog* OR ((functional* OR mental*) NEAR/2 (assess* OR status OR state)) OR EORTC OR "Rotterdam Symptom* Checklist*" OR rscl OR stress OR distress OR (("Short Form" OR sf) NEAR/1 (36 OR 20 OR 12 OR 8)) OR sf36 OR sf20 OR sf12 OR sf8 OR "Anderson Symptom* Inventor*" OR MDASI OR hads OR BCM20 OR "Brain Cancer Module")) )

**Google Scholar**

"brain|cerebral|intracranial tumor|tumour|neoplasms|metastases|malignancies" Radiosurgery|"gamma knife"|stereotactic "quality of life"|hrql|depression|"mood disorder"|affect|emotion|fatigue|"mental health"|wellbeing|"functional|mental assess|status|state"
